# Inter-observer and Intra-observer Agreement in Pathological Evaluation of Non-alcoholic Fatty Liver Disease Suspected Liver Biopsies

**DOI:** 10.5812/hepatmon.15167

**Published:** 2014-01-03

**Authors:** Omid Pournik, Seyed Moayed Alavian, Leila Ghalichi, Bahram Seifizarei, Leila Mehrnoush, Azam Aslani, Soghra Anjarani, Saeid Eslami

**Affiliations:** 1 Department of Medical Informatics, Faculty of Medicine, Mashhad University of Medical Sciences, Mashhad, IR Iran; 2 Faculty of Advanced Technologies in Medicine, Iran University of Medical Sciences, Tehran, IR Iran; 3 Middle East Liver Diseases Center (MELD), Tehran, IR Iran; 4 Baqiyatallah Research Center for Gastroenterology and Liver Diseases, Baqiyatallh University of Medical Sciences, Tehran, IR Iran; 5 Mental Health Research Center, Iran University of Medical Sciences, Tehran, IR Iran; 6 School of Medicine, Shahid Beheshti Hospital, Hamedan University of Medical Sciences, Hamedan, IR Iran; 7 Reference Health Laboratories Research Center, Tehran, IR Iran; 8 Pharmaceutical Research Center, School of Pharmacy, Mashhad University of Medical Sciences, Mashhad, IR Iran

**Keywords:** Fatty Liver, Observer Variation, Pathology

## Abstract

**Background:**

Histopathologic assessment of liver tissue is an essential step in management and follow-up of non-alcoholic fatty liver disease (NAFLD) while inter- and intra-observer variations limit the accuracy of these assessments.

**Objectives:**

The aim of this study was to assess the inter- and intra-observer reproducibility of histopathologic assessment of liver biopsies based on NAFLD activity score (NAS) scoring system.

**Materials and Methods:**

The anonymous liver biopsy samples of 100 consecutive NAFLD suspected adults were randomly assigned to four pathologists. Then, the samples were randomly reassigned to the pathologists for the second time in a way that each sample would be evaluated by two different pathologists. Biopsies were revisited by their first evaluator after two months. The results were reported based on NAS scoring system.

**Results:**

Inter-observer agreement of the pathology scores based on NAS scoring system was acceptable for steatosis, lobular inflammation, and fibrosis, but not for hepatocyte ballooning. The intra-observer agreement was acceptable in all scales, with lowest intra-class correlation observed for lobular inflammation.

**Conclusions:**

NAS scoring system has good overall inter- and intra-observer agreement, but more attention should be given to defining the hepatocyte ballooning and lobular inflammation, and training the pathologists to improve the accuracy of pathology reports.

## 1. Background

Considering the increasing prevalence of non-alcoholic fatty liver disease (NAFLD) world-wide, it is essential to have methods and procedures for accurate diagnosis of the cases, as well as to identify patients with non-alcoholic steatohepatitis (NASH) (1). The diagnosis of NASH and its distinction from non-alcoholic fatty liver (NAFL) affects the prognosis and treatment plan as the former group have a higher risk of acquiring cirrhosis and hepatocellular carcinoma (2).

Histopathologic assessment of liver tissue is an essential step in the management and follow-up of chronic liver conditions (3, 4). It is generally agreed that steatosis, hepatocellular ballooning, and lobular inflammation are the histopathological characteristics of NASH, while fibrosis is not essential in the diagnosis (2).

Although liver biopsy is the gold standard of staging and evaluating the progress of the disease, it is a risky procedure and there are a few limitations that affect the clinical acceptance of the process (1). Invasiveness is one of these factors which affects both patient and clinician tendency to the procedure (5), as well as patients compliance for repeated biopsies which might be necessary during the long-term management of the cases (6).

Sampling error is another issue which affects the acceptance of liver biopsy and it mainly results from sampling variability and observer variation. Sampling variability reflects the uneven distribution of histologic lesions in the liver tissue and may result in misdiagnosis and staging inaccuracy (5, 7). Inter- and intra-observer variation also limits the accuracy of the histopathologic evaluations and thus, affect the clinical judgment of the physicians (5, 6). This issue has been widely discussed in chronic liver disease especially viral hepatitis during the past 20 years, (8-10) and less frequently in biopsies obtained from NAFL-suspected cases. 

NAFLD comprises a wide morphological spectrum which makes the pathologic evaluation and distinction difficult. NAFLD is histologically further categorized into NAFL and NASH. The diagnosis is made based on the degree of steatosis, hepatocellular ballooning, and lobular inflammation (2). Histopathologic grading and staging of liver biopsies can be different between the pathologists (10, 11), and general pathologists and expert hepatopathologists can perform significantly different in assessing NASH suspected liver biopsies (12). Hepatocellular ballooning is highly prone to intra- as well as inter-observer variation (2), while the agreement is higher in fibrosis (13).

Interventions such as image review by the pathologists and use of scoring sheet with written diagnostic criteria for different NAFLD phenotypes have been applied to improve the agreement (14). Also, various scoring systems have been proposed to improve the agreement of the pathologists (12). NAFLD activity score (NAS) is a histologic scoring system, widely accepted for evaluating NASH suspected liver biopsies (15). NAS ranges between 0 and 8, and NAS scores of 0 to 2 are not considered as NASH; Scores of 3 to 4 are considered indeterminate and scores of 5 to 8 recommend NASH diagnosis. The primary studies in NASH Clinical Research Network (16) have shown acceptable inter- and intra-rater agreement between the pathologists, but there have been few studies to assess the validity of NAS scoring systems outside NASH Clinical Research Network (16, 17). 

Some researchers believe that NAS is a valuable tool in clinical trials, while its generalizability and diagnostic accuracy should be studied (18). Although histopathologic evaluation of liver biopsies using NAS is becoming a routine practice in clinic, its accuracy is not usually considered during clinical decision making in diagnosis and follow-up. Also, the degree of accuracy is not clear in settings other than NASH Clinical Research Network. In this study, we intended to assess both inter-observer and intra-observer reproducibility of NAS scoring system in a group of Iranian pathologists.

## 2. Objectives

The aim of this study was to help the clinicians better judge the results of the liver biopsies both on diagnosis and follow-up of NAFLD patients.

## 3. Materials and Methods

In this cross-sectional study, 100 consecutive liver samples of adult cases suspected to NAFLD whom were biopsied in 6 different hospitals in Tehran, Iran, between 2010 and 2012 were included. All of the cases were diagnosed for NAFLD based on clinical evaluation and evidence of steatosis in ultrasonography after ruling out the other etiologies of fatty liver like excessive alcohol consumption and other chronic liver disease. The cases were Iranian and older than 18 years old.

Ultrasound-assisted percutaneous liver biopsy was performed using Tru-Cut biopsy needles.

The biopsy samples were sectioned in 3 different levels and stained by hematoxylin and eosin, trichrome, and reticulin methods. For the purpose of the study, these anonymous biopsy section samples were randomly assigned to four pathologists (3 general pathologists and one hepatopathologist) who had agreed to evaluate the samples. The pathologists were not aware of patient identity or open label pathology report, and reported the samples based on NAS scoring system (15). The samples were excluded from the study if the observers reported inadequate quality for the biopsy or staining.

When all samples were evaluated, they were randomly reassigned to the pathologists for the second time in a way that each sample would be evaluated by two different pathologists. The pathologists were blinded to the result of the prior evaluation and the identity of the first evaluator. The agreement between the 2 raters was evaluated by intra-class correlation (ICC). 

From 100 biopsies, 91 cases were revisited by their first evaluator after 2 months and reported based on NAS scoring system. The agreement between the 2 evaluations was evaluated by ICC to assess the intra-observer agreement.

The study was approved by the ethical committee of Middle East Center of Liver Disease (MELD). The results were analyzed using (SPSS version 16, Chicago, IL) Mean and standard deviation were used to describe the data. ICC was applied to assess the correlation between the pathologists' evaluations in different scales. 

## 4. Results

One hundred liver biopsies of adult cases were evaluated. Four cases were excluded due to technical problems in staining. The mean age of participants was 41.5 (SD: 9.74) years and the range was 18 to 58 years. Sixty four cases were male.

The results of the NAS total score based on the first pathologists' assessment is presented in Table 1. 

**Table 1.** Results of Total NAFLD Activity Score, Scores Based on the First Pathologists' Evaluation ^a^

NAFLD Activity Score	Samples, No.
**0–2**	35
**3–4**	30
**5-8**	31

^a^According to NAS system, if NAS score was 0-2 the diagnosis was NAFL; if NAS score was 3-4 the diagnosis was indeterminate; if NAS score was 5-8 the diagnosis was NASH.

The ICC of the total NAS scores and steatosis, lobular infiltration, and ballooning scores are presented in Table 2. The ICC for total NAS score, steatosis, and fibrosis was more than 0.5. For lobular inflammation, the ICC was low but significant. The Inter-observer ICC for hepatocyte ballooning was not acceptable. 

**Table 2.** Inter-Observer Agreement of the Pathology Scores Based on NAS Scoring

	ICC ^a^	95% confidence Interval	P value
**Steatosis**	0.654	0.523-0.755	< 0.001
**Lobular inflammation**	0.288	0.095-0.461	0.002
**Hepatocyte Ballooning**	0.012	-0.187-0.211	0.452
**Total score**	0.623	0.484-0.731	< 0.001
**Fibrosis**	0.504	0.338-0.639	< 0.001

^a^Abbreviations: ICC, intra-class correlation.

The intra-observer agreement of the pathologists in different scales is demonstrated in Table 3. All scales showed significant level of ICC. Lowest intra-observer ICC was observed for lobular inflammation and the highest was observed for steatosis. There was no significant difference in the intra-observer agreement of the pathologists. 

**Table 3.** The Intra-Observer Agreement of the Pathology Scores Based on NAS Scoring

	ICC ^a^	95% confidence Interval	P value
**steatosis**	0.754	0.648-0.831	< 0.001
**Lobular inflammation**	0.420	0.234-0.577	< 0.001
**Hepatocyte Ballooning**	0.563	0.403-0.690	< 0.001
**Total score**	0.686	0.558-0.782	< 0.001
**Fibrosis**	0.744	0.632-0.836	< 0.001

^a^ Abbreviation: ICC, intra-class correlation.

## 5. Discussion

In our study, highest agreement was observed in steatosis scale both in inter-observer and intra-observer assessments. The agreement for hepatocyte ballooning and lobular inflammation was lower compared to other scales. Research has shown that ballooning and lobular inflammation are important pathologic features that enable discrimination of NASH (19). The study of Kleiner et al. (15) showed that the agreement on ballooning feature was acceptable although the agreement was low in inter-rater agreement of pediatric cases (kappa = 0.22). Fukusato et al. who evaluated inter-rater agreement of experienced hepatopathologists observed slight or poor agreement in steatosis, ballooning, intralobular necroinflammatory changes, and portal inflammation (20). Some experts believe that hepatocellular ballooning is “an ill-defined form of liver cell injury associated with cell swelling and rounding of the cytoplasm, the detection of which is prone to intra- as well as inter-observer variation”(2).

We observed moderate inter-rater agreement and good intra-rater agreement in fibrosis score, while Kleiner et al. (15) showed highest agreement in this feature compared to other scales, although fibrosis is not essential in the diagnosis of NASH (2).

Previous studies have shown lower agreement in pediatric cases compared to adults (11, 15). Our cases were at least 18 years and no comparison could be provided.

Variability in the distribution of hepatic lesions within the liver reduces the accuracy of liver biopsy as the gold standard (21). Considering the mentioned weak points, some researchers suggest considering liver biopsy as the best rather than gold standard accurate staging and grading of chronic liver conditions (22).

Experts have suggested a few methods for reducing the risk of misclassification. Interpretation of biopsies by experienced liver pathologists is one of these methods (22). Others have proposed that evaluation of hepatocellular ballooning might be more accurately performed by immunostaining methods (2). Providing clinical and laboratory findings of the patients could also be helpful in correct evaluation of the patients by the pathologists (23).

Despite the importance of histopathologic findings in the management of liver diseases and their increasing prevalence, most of the pathologists have not received enough formal training and have little experience with liver biopsy (23). 

Some researchers have shown that in evaluation of liver samples from chronic viral hepatitis, the level of experience of the pathologist in terms of specialization, duration, and location of practice has a stronger effect on the agreement compared to the characteristics of the specimen (10). 

Many non-invasive methods have been proposed for evaluation of NAFLD–suspected cases. Fibroscan® is one of these methods which has gained popularity in the clinic. Accuracy of Fibroscan® is variable especially in health conditions such as steatosis, metabolic syndrome, high body mass index, hepatic hemangioma, and heart failure (24). Another limitation of Fibroscan® is based on the fact that the results are displayed as a single score and the clinicians do not have the chance to evaluate different sub-scales which are present in routine histopathologic reports.

Although many experts believe that histological assessment of liver in NAFLD cases “is far from being both accurate and precise” (1), it is still the best available and acceptable method for evaluation and follow up of NAFLD cases. The downsides of the procedure are to be considered with attention and ancillary methods should be applied to improve the accuracy and reduce the risks. Development and improvement of scoring systems and complementary educational programs for pathologists are among possible solutions for improving the accuracy. Future technical developments may lead to new and improved methods and higher precision in laboratory and clinical evaluation of NAFLD patients.

Finally, NAS scoring system has good overall inter-observer and intra-observer agreement, but more attention should be paid both in defining the hepatocyte ballooning and lobular inflammation and training of the pathologists to improve the accuracy of the pathology reports.
